# Protic Imidazolium Polymer as Ion Conductor for Improved Oxygen Evolution Performance

**DOI:** 10.3390/polym11081268

**Published:** 2019-07-31

**Authors:** Fangfang Zhang, Minchen Yang, Siyi Zhang, Pengfei Fang

**Affiliations:** Hubei Nuclear Solid Key Laboratory, College of Physics and Science Technology, Wuhan University, Wuhan 430072, China

**Keywords:** oxygen evolution, ion conductor, imidazolium, electrocatalysis, electrode

## Abstract

Improving the electrocatalytic performance of oxygen evolution reaction (OER) is essential for oxygen-involved electrochemical devices, including water splitting and rechargeable metal–air batteries. In this work, we report that the OER performance of commercial catalysts of IrO_2_, Co_3_O_4_, and Pt-C can be improved by replacing the traditional Nafion^®^ ionomer with newly synthesized copolymers consisting of protonated imidazolium moieties such as ion conductors and binders in electrodes. Specifically, such an improvement in OER performance for all the tested catalysts is more significant in basic and neutral environments than that under acidic conditions. We anticipate that the results will provide new ideas for the conceptual design of electrodes for oxygen-involved electrochemical devices.

## 1. Introduction

The development of advanced energy storage and conversion technologies is essential to cope with the increasing environmental pollution and the global energy crisis caused by the depletion of fossil fuels [1,2]. Of various technologies developed, oxygen-involved electrochemical energy storage and conversion devices, including fuel cells and rechargeable metal–air batteries, have attracted extensive attention due to their high power and energy density [3,4,5,6,7]. Oxygen evolution reaction (OER) has been recognized as one of the most important reactions for electrochemical energy technologies. The typical electrode for OER consists of electrocatalysts or electron-conductive materials-supported electrocatalysts and ion-conductive polymers. Although extensive works have focused on the development of advanced electrocatalysts to reduce the overpotential of the oxidation of adsorbed oxygenated species at the surface of electrocatalysts for the generation of oxygen molecules during the OER process [8,9,10,11], the effect of ion-conductive polymers on the performance of OER should not be neglected.

Ion-conductive polymers in electrodes for oxygen-involved electrochemical reactions function not only as ion conductors but also as binders to ensure the mechanical stability of the catalyst layers and to improve the interactions between electrolytes and electrocatalysts [12,13,14,15,16]. The often-applied ion-conductive polymer in electrodes is perfluoronated sulfonic acid (PFSA) ionomers, typically Nafion^®^ from Dupont. The introduced PFSA ionomers are generally covered on the surface of catalysts through electrostatic interactions, forming an ion-conductive layer [17,18]. However, the relatively low solubility and diffusivity of oxygen in PFSA layers could constrain the electrode reaction kinetics due to the resistance of oxygen transport on or near the surface of electrocatalysts [19]. Thus, the development of new ion-conductive polymers applied to the electrocatalyst layer to reduce the resistance of oxygen transport could be an effective approach to enhance the electrocatalytic activity of oxygen-involved electrochemical reactions.

Room temperature ionic liquids (RTILs), also known as molten salt, have been widely applied in the areas of electrochemistry due to their unique properties, including low volatility, good chemical stability, high ionic conductivity, and wide electrochemical window [20,21,22,23]. Moreover, imidazolium-type RTILs exhibit good oxygen solubility and diffusivity [24,25,26]. Therefore, RTILs have been introduced to electrodes as additional additives to improve the electrocatalytic activity towards oxygen-involved reactions, including OER and oxygen reduction reactions (ORR) [27,28,29]. However, the leaching of small RTIL molecules and the interaction of ionic segments with sulfonate acid groups on PFSA can limit their functions in electrodes. This has inspired researchers to synthesize new polymers containing protonated imidazolium segments as an alternative ion conductor to replace PFSA ionomers in oxygen electrodes. For example, Yan et al. reported that the oxygen reduction reaction activity of Pt/C can be significantly improved by replacing Nafion with a tertiary ammonium-type protic poly(ionic liquid)s as a proton conductor [30]. Different types of poly(ionic liquid) have been synthesized, as reported in the recent topical review; however, the applications were mainly focused on ion conductive membranes and gas separation [31,32].

In this work, we report the OER performance of commercially available catalysts of IrO_2_, Co_3_O_4_, and Pt/C under acidic, basic, and neutral conditions using the newly synthesized copolymers containing styrene and protonated imidazolium segments as ion conductors and binders. It is expected that protonated imidazolium can provide excellent ion conductivity and a relatively weak interaction with electrocatalysts, thus improving their catalytic activity towards OER. This work may provide new ideas for design of air electrode with widespread applications.

## 2. Materials and Methods

### 2.1. Materials

1-Vinylimidazole (Vim), Bis(trifluoromethane)sulfonamide (HTFSI), and diethyl ether were purchased from Alfa Aesar (Tianjin, China) and used as received. Toluene (Sinopharm, China) was distilled over sodium under reduced pressure using benzophenone as indicator. 2,2′-Azobisisobutyronitrile (AIBN, Sinopharm, China) was recrystallized in ethanol. Styrene (Sty, Alfa Aesar) was purified through a basic alumina column to remove inhibitor. Pt/C catalyst (20 wt %, Hispec 3000) was purchased from Hesen Electric Co (Shanghai, China). Co_3_O_4_ (99%) was supplied by Alfa Aesar (Tianjin, China). IrO_2_ (85%) was purchased from Kaida Chemical Engineering Company (Shanxi, China). Water was deionized using a Ulupure-H ultrapure water generator (Ulup, China) with resistivity of 18.2 MΩ cm^−1^.

### 2.2. Synthesis of Poly(1-vinylimidazole-co-styrene) (PS-b-PVIm)

Desired amounts of 1-vinylimidazole, styrene, and AIBN were dissolved in anhydrous toluene under nitrogen atmosphere in a Schlenk flask. After three freeze-pump-thaw cycles to remove dissolved oxygen, the mixture was placed in a water bath pre-heated to 60 ± 1 °C for polymerization of 12 h under protection of nitrogen. The resulting solution was then dropped into cold diethyl ether, and precipitates were collected by filtration. After being washed five times with cold diethyl ether, the product was dried under vacuum at 80 °C for 24 h. The detailed amounts of added chemicals were listed in Table 1.

### 2.3. Protonation of PS-b-PVIm

The above-synthesized copolymer and excess HTFSI were dissolved in ethanol, and the mixture was stirred for 12 h using magnetic stirrer bar. The solution was then dropped into deionized water, and the precipitate was collected by filtration. After being washed five times with deionized water, the product was dried under vacuum at 40 °C for 24 h and denoted as PS_1_-b-P(VImH^+^)_n_TFSI (where n refers to the molar ratio of initially added molar fraction of 1-vinylimidazole with respect to styrene). Prior to application for OER, the synthesized product was dissolved in a mixed solvent of isopropyl alcohol and deionized water with a volume ratio of 4/1 to form 5 wt % PS_1_-b-P(VImH^+^)_n_TFSI solution.

### 2.4. Characterizations

Fourier transform infrared (FTIR) spectra were recorded on NEXUS 670 spectrometer (Nicolet, USA) with a resolution of 4 cm^−1^ to determine chemical composition of the synthesized copolymer. 1H nuclear magnetic resonance (^1^H NMR) was applied to investigate the chemical structure of products on Ascend400 NMR spectrometer (Bruker, CH) using tetramethylsilane as internal standard and *d*_6_-DMSO as deuterated solvent. Elemental analysis was carried out on a Vario MACRO Cube analyzer (Elementar, Germany) to determine the monomeric ratio and the degree of protonation of the synthesized copolymers.

### 2.5. Electrochemical Measurements

All electrochemical measurements are performed on an electrochemical workstation (CHI604D) with a rotating system (PINE research, PHYCHEMI). The electrocatalytic performance was characterized by linear sweep voltammetry (LSV, 1600 rpm with scan rate of 5 mV s^−1^) and chronopotentionmetry in a standard rotating disk electrode at ambient temperature. Electrochemical impedance spectroscopy (EIS) was carried out at 0.45 V (RHE) in N_2_-staturted HClO_4_ with rotating speed at 2500 rpm to measure the protonic resistance. A typical three-electrode system was applied using catalyst coated glassy carbon (GC, 0.196 cm^2^) as working electrode, 1 cm^2^ Pt coil as counter electrode, and saturated calomel electrode (0.1 M HClO_4_ or phosphate buffer saline (PBS, pH = 7) as electrolyte) or Hg/HgO electrode (0.1 M Potassium hydroxide solution(KOH) as electrolyte) as reference electrode. To fabricate working electrode, 4 mg commercial catalyst (Pt/C, IrO_2_, or Co_3_O_4_), 20 μL ionomer dispersion (5 wt % Nafion^®^ or 5 wt % PS_1_-b-P(VImH^+^)_n_TFSI), 800μL isopropyl alcohol, and 200 μL of deionized water were mixed, and the mixture was ultrasonicated in an ice bath for 30 min to form a homogenous catalyst ink. Afterwards, 10 μL well-dispersed catalyst ink was dropped onto GC disk and the electrode was dried in air. The catalyst loading was about 200 μg cm^−2^. Prior to the measurement, the electrolyte was saturated with oxygen for 30 min.

## 3. Results and Discussion

### 3.1. Material Properties

**Figure 1:**
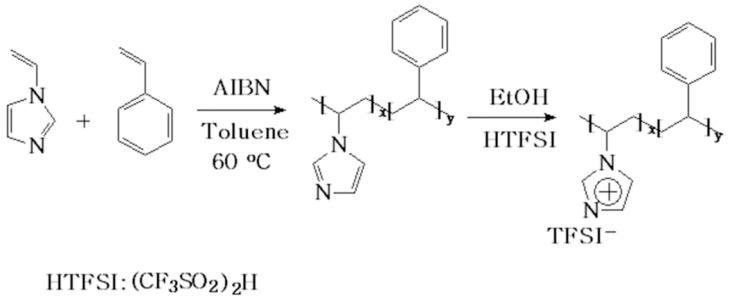
Synthetic route of model copolymer of poly(1-vinylimidazole-co-styrene) PS-b-P(VImH^+^)TFSI.

The motivation of this work is to understand whether protonated imidazolium polymers can replace PFSA ionomers as ion conductors and binders for enhanced OER performance. Thus, we first designed copolymers consisting of protonated imidazolium segments as ion transport media and polystyrene segments providing mechanical strength and hydrophobic environment. The model copolymers were synthesized by a simple free radical polymerization initiated by AIBN using mixed monomer solutions of 1-vinylimidazole and styrene, followed by protonation of the copolymer using HTFSI as proton donor, as shown in Figure 1. It has been theoretically proposed that medium fraction of ionic groups is suitable for hydrocarbon ionomers applied in electrode, since low fraction of ionic groups cannot provide sufficient ion conductivity and high fraction of ionic groups leads to difficulty in permeation of oxygen [33]. Thus, copolymers with different protonated imidazolium segments were synthesized in this work.

**Figure 2:**
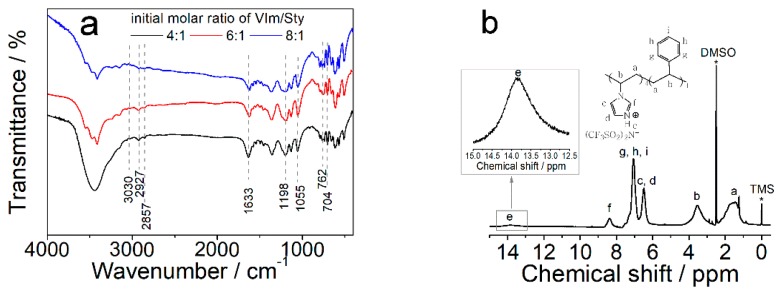
(**a**) Fourier transform infrared (FTIR) spectra of PS_1_-b-P(VImH^+^)_n_TFSI with different molar ratio of initially added monomers (VIm/Sty). (**b**) ^1^H NMR spectrum of PS_1_-b-P(VImH^+^)_4_TFSI.

FTIR spectra were first recorded to qualitatively confirm the successful synthesis of PS_1_-b-P(VImH^+^)_n_TFSI ionomers, as shown in Figure 2a. The observed absorption band at 1633 cm^−1^ is a typical indicator for complexation of C=N groups with electron-deficient moieties, suggesting the successful protonation of imidazole moieties by HTFSI [34]. The absorption bands at 1198 and 1055 cm^−1^ are assigned to the deformation vibration of S=O groups in TFSI. The C–H out-of-plane bending vibrations of benzene ring were observed at absorption bands of about 762 and 704 cm^−1^. The stretching vibration bands of unsaturated methylene groups in aromatic rings and the methylene as well as methyne groups in the polymer main chain appeared at 3030, 2927, and 2857 cm^−1^, respectively. ^1^H-NMR spectrum (Figure 2b) further confirmed the protonation of imidazolium moieties, as evidenced by the appeared resonance peak at around 13.8 ppm corresponded to the transferred proton from HTFSI to the N-atom on imidazole rings.

To quantitively determine the chemical composition and degree of protonation of the synthesized copolymers, elemental analysis was conducted since only imidazole moieties contains nitrogen atoms and TFSI^－^ has sulfur atoms in the synthesized ionomers. The original results of elemental analysis and the calculated molar ratios of different segments in copolymers are listed in Table 2. It is apparent that with the increase in the fraction of VIm in monomer solutions, the corresponding molar ratio of VIm and Sty segments in the formed copolymers increased. However, the molar ratio of VIm and Sty segments is quite different from the initially added monomer ratio due to the high polymerization reactivity of styrene [35].

### 3.2. Electrocatalytic Activity

**Figure 3:**
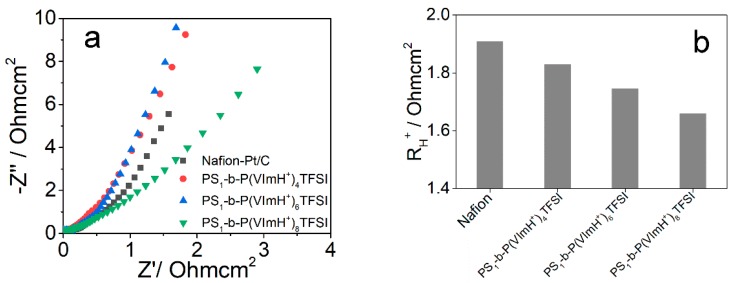
(**a**) Nyquist plots of impedance spectra measured under N_2_-saturated 0.1 M HClO_4_ for different catalyst layers. (**b**) The proton resistance R_H_^+^ of different catalyst layers calculated from (**a**).

In typical electrodes for OER, sufficient ion conductivity is essential for the electrocatalytic reactions. Huang et al. recently reported that the proton resistance in catalyst layer can be calculated by the intercept of the linear portion on the *x*-axis in electrochemical impedance spectrum (EIS) [36]. Thus, we recorded the EIS spectra of Pt/C catalysts using the synthesized copolymers as well as Nafion^®^ as proton conductors and binders in nitrogen-saturated 0.1 M HClO_4_ aqueous solutions, as shown in Figure 3a. The accordingly calculated proton resistance (R_H_^+^) normalized to the area of catalyst layers was displayed in Figure 3b. It is evident that R_H_^+^ values of all the synthesized copolymers are smaller than that of Nafion^®^, indicating the improved proton conductivity in electrode. Moreover, R_H_^+^ decreased from 1.83 to 1.66 ohmcm^2^ with the increase in the ratio of PS and VIm from 1:4 to 1:8 due to the increased ionic fraction in the synthesized copolymers.

**Figure 4:**
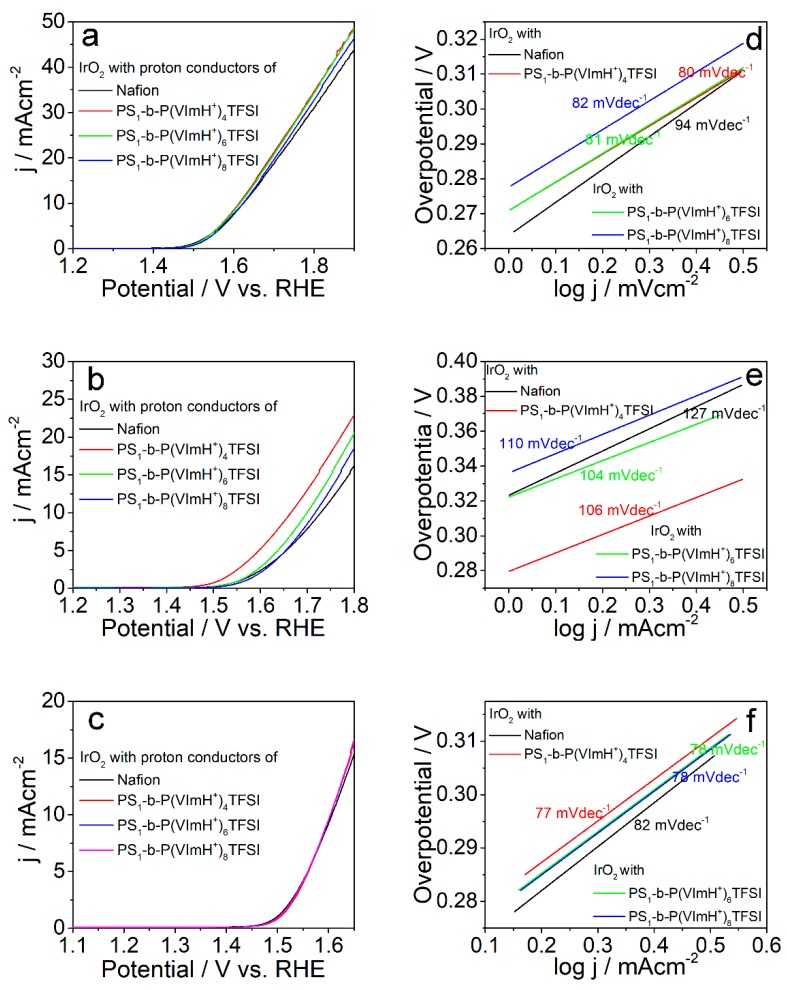
(**a**–**c**) Linear sweep voltammetry (LSV) curves and (**d**–**f**) Tafel plots of IrO_2_ catalyst using different ionic conductors, as indicated in the figures with a sweep rate of 5 mVs^−1^ in (**a**,**d**) 1.0 M KOH, (**b**,**e**) PBS buffer, and (**c**,**f**) 1.0 M HClO_4_.

The electrocatalytic activity of commercial catalyst of IrO_2_ towards OER using PS_1_-b-P(VImH^+^)_n_TFSI as ion conductor and binder was investigated under basic, neutral, and acidic conditions using linear sweep voltammetry (LSV) analysis, as shown in Figure 4a–c. For comparison, LSV curves using Nafio^®^ ionomers were plotted in the same figures. Compared with Nafion^®^/IrO_2_ catalysts, improvements in OER catalytic activity under basic and neutral conditions were observed for PS_1_-b-P(VImH^+^)_n_TFSI/IrO_2_ catalysts whereas the OER activity in acidic conditions is comparable to Nafion^®^/IrO_2_ catalysts, as evidenced by the change of overpotential at 10 mAcm^−2^. For instance, the overpotentials of PS_1_-b-P(VImH^+^)_4_TFSI/IrO_2_ at 10 mAcm^−2^ are about 13 mV and 64 mV less than those of Nafion^®^/IrO_2_ in basic and neutral conditions, respectively. It has been reported that imidazolium moieties has higher oxygen solubility and transport rate compared with sulfonic acid groups [25,33,37], which might be the reason for the improved OER performance after replacing Nafion^®^ with PS_1_-b-P(VImH^+^)_n_TFSI as ion conductor. In addition, the OER activity decreased with the increase in the content of protonated imidazolium moieties in the synthesized copolymers, although ionic conductivity increased. This could mean that the decreased hydrophobicity of microenvironment induced by the increase in protonated imidazolium moieties is unfavorable for transport of the generated oxygen molecules, leading to the decreased catalytic activity towards OER [26,38]. This result demonstrates that the hydrophobicity of microenvironment near the surface of catalysts has significant influence on the catalytic activity if the ionic conductivity is sufficient.

To elucidate the mechanism of OER for the PS_1_-b-P(VImH_+_)_n_TFSI/IrO_2_ catalysts, Tafel plots, which refer to the linear portions of the polarization curve in the low potential regions, were displayed in Figure 4d,e. It is evident that the Tafel slopes for PS_1_-b-P(VImH^+^)_n_TFSI/IrO_2_ catalysts under all the tested conditions are smaller than that for Nafion^®^/IrO_2_, indicating the more efficient OER performance for PS-b-PS_1_(VImH^+^)_n_TFSI/IrO_2_ compared to Nafion^®^/IrO_2_. Moreover, the calculated Tafel slopes as indicated in the figure suggest that the adsorption of oxygenated species on the surface of catalysts is the rate-determining step for OER [39,40].

**Figure 5:**
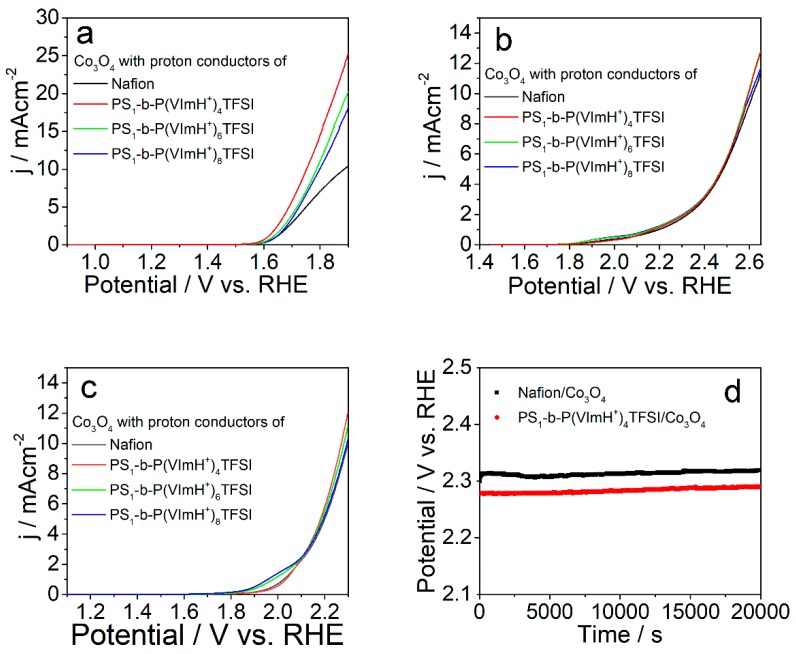
LSV curves of Co_3_O_4_ catalyst using different ion conductors as indicated in the figures with a sweep rate of 5 mVs^−1^ in (**a**) 1.0 M KOH, (**b**) phosphate buffer saline (PBS) buffer, and (**c**) 1.0 M HClO_4_. (**d**) Chronopotentiometric analysis of the catalyst stability for mixed Co_3_O_4_ catalyst with Nafion^®^ and PS_1_-b-P(VImH^+^)_4_TFSI at 10 mAcm^−2^ in 0.1 M HClO_4_ solutions.

Despite the fact that the catalyst loading and the overall architecture of the two electrodes are very similar, the OER performance of the two electrodes is quite different. To bring more insight into this phenomenon, two additional works were carried out using commercial Co_3_O_4_ and Pt-C as OER catalysts. Figure 5 displays LSV curves of PS_1_-b-P(VImH^+^)_n_TFSI/Co_3_O_4_ with different initial molar ratio of two monomers under various conditions. Compared with Nafion^®^/Co_3_O_4_ catalyst, all the PS_1_-b-P(VImH^+^)_n_TFSI/Co_3_O_4_ samples exhibited an improved OER performance under all the tested conditions. Particularly, the overpotential was negatively shifted by 134 mV for PS_1_-b-P(VImH^+^)_4_TFSI/Co_3_O_4_ sample with respect to Nafion^®^/Co_3_O_4_ catalyst in 0.1 M KOH aqueous solution (Figure 5a), whereas the OER performance was slightly higher than that of Nafion^®^/Co_3_O_4_ catalyst in neutral and acidic conditions (Figure 5b,c). Again, the higher oxygen solubility and oxygen transport rate after replacing Nafion^®^ with PS_1_-b-P(VImH^+^)_4_TFSI as ion conductor as well as the enhanced ionic conductivity were responsible for the improved OER performance. Particularly, the basic condition makes the adsorption of oxygenated species (Oad) at the surface of catalysts was more favorable compared with neutral or acidic conditions, resulting in more pronounced improvement of OER performance in basic condition [41,42,43]. In addition, stability of the designed catalyst was evaluated by chronopotentiometric analysis at a constant current density of 10 mAcm^−2^ in 0.1 M HClO_4_ solution using PS_1_-b-P(VImH^+^)_4_TFSI/Co_3_O_4_ as an example, as shown in Figure 5d. It is apparent that the potential to maintain 10 mAcm^−2^ increased slightly by 12 mV for Nafion^®^/Co_3_O_4_ and by 19 mV for PS_1_-b-P(VImH^+^)_4_TFSI/Co_3_O_4_ within the tested 20,000 s, respectively. However, the stability of PS_1_-b-P(VImH^+^)_4_TFSI/Co_3_O_4_ is comparable to Nafion/Co_3_O_4_. Moreover, the potential fluctuated due to the accumulation of oxygen bubbles on the catalyst surface, which was hard to be discharged. Although hydrocarbon-type poly(ionic) liquids have been reported as less stable than Nafion while applied as proton conductors for oxygen reduction reactions of Pt/C catalysts [30], this seems not to be the case for oxygen evolution reactions in this study, probably due to the difference in the generated intermediates of active oxygen species. This needs to be further investigated and will be reported in subsequent publications.

**Figure 6:**
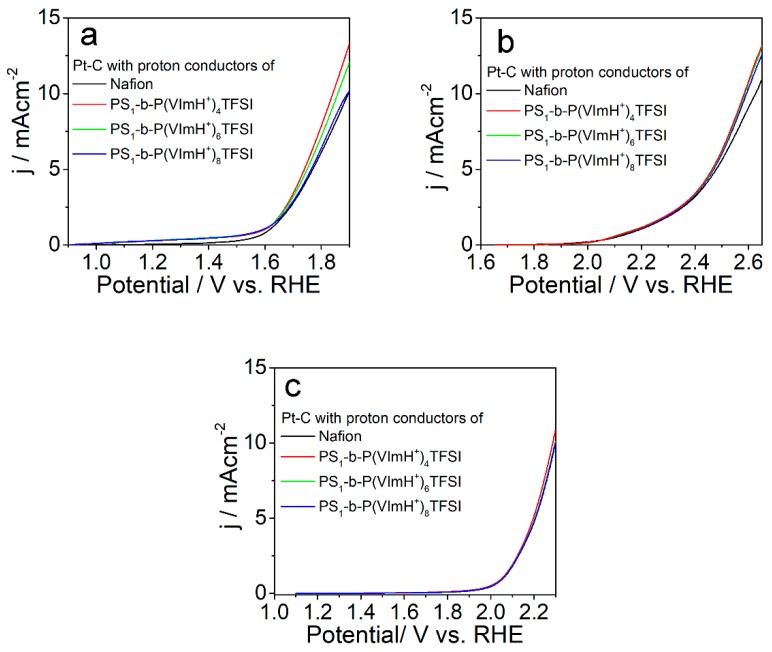
LSV curves of Pt-C catalyst using different ion conductors as indicated in the figures with a sweep rate of 5 mVs^−1^ in (**a**) 1.0 M KOH, (**b**) PBS buffer, and (**c**) 1.0 M HClO_4_.

As for commercial Pt-C catalyst, similar phenomena were observed while replacing Nafion^®^ with the synthesized copolymers of PS_1_-b-P(VImH^+^)_n_TFSI, as shown in Figure 6. Of the synthesized copolymers, PS_1_-b-P(VImH^+^)_4_TFSI/Pt-C exhibited the best OER performance under all the tested conditions (basic, neutral, and acidic environments). The potential for generation of 10 mAcm^−2^ current using PS_1_-b-P(VImH^+^)_4_TFSI/Pt-C catalyst negatively shifted by about 60 mV compared with Nafion^®^/Pt-C catalyst under 0.1 M KOH solution, whereas the negative shifts were about 45 mV and 16 mV under neutral PBS buffer solution and 0.1 M HClO_4_ solution, respectively, indicating that the improvement in OER performance by using PS_1_-b-P(VImH^+^)_n_TFSI as ion conductor was more pronounced in a basic environment than that under neutral and acidic conditions. With the observed results of OER performance, we can conclude that introduction of ionomers containing protonated imidazolium moieties as ion conductor can improve OER performance compared with catalysts using traditional Nafion as ion conductors due to the enhanced ionic conductivity and the facilitated oxygen transport.

## 4. Conclusions

Copolymers containing protonated imidazolium segments were successfully synthesized through simple free radical polymerization and were introduced into electrodes as ion conductors and binders for electrocatalytic oxygen evolution reaction (OER). The synthesized copolymers exhibited better ionic conductivity in electrodes compared to the often applied Nafion^®^ ionomers. After replacing Nafion^®^ ionomers with the synthesized copolymers, the OER actvity of three commercially available catalysts of IrO_2_, Co_3_O_4_, and Pt-C improved, particularly in basic and neutral environments, and the stability of catalysts combined with the synthesized copolymers was comparable to catalysts associated with Nafion^®^ ionomers. Such improvement in OER performance is attributed to the enhanced ionic conductivity and improved oxygen solubility and permeation in ionomer layers at/near the surface of catalysts. The results demonstrate that replacing Nafion^®^ ionomers with rational ionomers could be an effective way to design and make effective electrodes for oxygen-involved electrochemical devices.

## Figures and Tables

**Table 1:** Ingredients for free radical polymerization.

Molar Ratio of VIm/Sty	Vim (mL)	Sty (mL)	Toluene (mL)	AIBN (mg)
4:1	36.20	13.16	60.72	0.048
6:1	54.29	13.16	84.50	0.067
8:1	72.39	13.16	108.28	0.086

**Table 2:** Elemental analysis and the calculated molar ratio of different segments for PS_1_-b-P(VImH^+^)_n_TFSI polymerized from different mixed monomer solutions.

VIm:Sty:HTFSI in Feed (Molar Ratio)	Elemental Analysis Results (wt%)				VIm:Sty:TFSI in Ionomer (Molar Ratio)
	C	H	N	S	
4:1:4	47.05	1.95	7.72	11.19	1.0:1.75:1.0
6:1:6	36.04	1.69	9.00	13.81	1.1:1.0:1.1
8:1:8	34.19	1.73	9.40	14.20	1.4:1.0:1.5
